# Association between liver-type fatty acid-binding protein and hyperuricemia before and after laparoscopic sleeve gastrectomy

**DOI:** 10.3389/fendo.2022.993137

**Published:** 2022-10-06

**Authors:** Hui You, Huihui Ma, Xingchun Wang, Xin Wen, Cuiling Zhu, Wangjia Mao, Le Bu, Manna Zhang, Jiajing Yin, Lei Du, Xiaoyun Cheng, Haibing Chen, Jun Zhang, Shen Qu

**Affiliations:** ^1^ Department of Endocrinology and Metabolism, Shanghai Tenth People’s Hospital, School of Medicine, Tongji University, Shanghai, China; ^2^ Shanghai Center of Thyroid Diseases, Shanghai Tenth People’s Hospital, School of Medicine, Tongji University, Shanghai, China; ^3^ Research Center for Translational Medicine at East Hospital, Tongji University School of Medicine, Tongji University, Shanghai, China; ^4^ Shanghai Institute of Stem Cell Research and Clinical Translation, Shanghai, China

**Keywords:** liver-type fatty acid-binding protein, laparoscopic sleeve gastrectomy, hyperuricemia, obesity, uric acid

## Abstract

**Background:**

Liver-type fatty acid-binding protein (FABP1) contributes to metabolic disorders. However, the relationship between FABP1 and hyperuricemia remains unknown. We aimed to evaluate the correlation between serum FABP1 and hyperuricemia in patients with obesity before and after laparoscopic sleeve gastrectomy (LSG).

**Methods:**

We enrolled 105 patients (47 men and 58 women) with obesity who underwent LSG. They were divided into two groups: normal levels of uric acid (UA) (NUA, n = 44) and high levels of UA (HUA, n = 61) with matching sexes. FABP1 levels and other biochemical parameters were measured at baseline and 3, 6, and 12 months after LSG.

**Results:**

Serum FABP1 levels were significantly higher in the HUA group than in the NUA group (34.76 ± 22.69 ng/mL vs. 25.21 ± 21.68 ng/mL, *P*=0.024). FABP1 was positively correlated with UA (r=0.390, *P*=0.002) in the HUA group. The correlation still existed after adjusting for confounding factors. Preoperative FABP1 levels were risk factors for hyperuricemia at baseline. UA and FABP1 levels decreased at 3, 6, and 12 months postoperatively. FABP1 showed a more significant decrease in the HUA group than in the NUA group at 12 months (27.06 ± 10.98 ng/mL vs. 9.54 ± 6.52 ng/mL, *P*=0.003). Additionally, the change in FABP1 levels positively correlated with changes in UA levels in the HUA group 12 months postoperatively (r=0.512, *P*=0.011).

**Conclusions:**

FABP1 was positively associated with UA and may be a risk factor for hyperuricemia in obesity. FABP1 levels were higher but decreased more after LSG in obese patients with hyperuricemia than in those without hyperuricemia.

## Introduction

Uric acid (UA) is a final enzyme product of purine nucleotide degradation and can scavenge oxygen radicals and protect the erythrocyte membrane from lipid oxidation (1). Hyperuricemia is a metabolic disease characterized by elevated serum UA (SUA) concentration (>420 μmol/L for men and >360 μmol/L for women) (2). In the past few decades, the prevalence of hyperuricemia has been increasing rapidly worldwide (2). In addition, hyperuricemia has been traditionally considered a potential risk factor for obesity, diabetes mellitus, nonalcoholic fatty liver disease (NAFLD), and other metabolic syndromes (3, 4). However, the underlying mechanisms of hyperuricemia and its risk factors remain unclear.

Liver-type fatty acid-binding protein (FABP1) (5) is a member of the fatty acid-binding protein family and is distributed mainly in the liver, kidney, lung, and gastrointestinal tract, accounting for 3% to 5% of the total cytoplasmic protein. It mainly enhances long-chain fatty acid uptake and fatty acid metabolism, and plays a central role in the hepatic β-oxidation of unesterified fatty acids, ultimately leading to various metabolic disorders (5–8). A previous study (8) has found that FABP1 can play a negative regulatory role in the formation of very-low-density lipoproteins, which can lead to lipid metabolism disorders when combined with total cholesterol, triglycerides, and apolipoprotein B, thereby accelerating NAFLD progression. Tsai et al. (9) found increasing concentrations of FABP1 were associated independently and significantly with diabetic nephropathy. Our previous research (10) also found serum FABP1 levels were correlated closely with obesity. However, the link between FABP1 and hyperuricemia has not been reported.

Recently, laparoscopic sleeve gastrectomy (LSG) (1, 11) has become a popular procedure to assist with weight loss owing to its low damaging effects on the patient’s physiology, high safety, and evident curative effect. This procedure has been shown to improve many metabolic syndromes, such as obesity, diabetes, and NAFLD, as well as hyperuricemia (12). Therefore, we aimed to evaluate the correlation between serum FABP1 and hyperuricemia in patients with obesity before and after LSG.

## Methods

### Patients

This retrospective, follow-up study enrolled 105 patients with obesity (body mass index [BMI], 38.39 ± 6.33 kg/m^2^) aged 18 to 60 years from the Shanghai Tenth People’s Hospital. Patients were divided into the following two groups according to their SUA levels (1): normal levels of UA (NUA, men ≤ 420 μmol/L, women ≤ 360 μmol/L, n = 44) and high levels of UA (HUA, men > 420 μmol/L, women > 360 μmol/L, n = 61) groups. There was no statistical difference in sex distribution between the two groups. All patients underwent LSG. Exclusion criteria were patients with: (1) a history of substance abuse or chronic mental illness; (2) psychiatric disorders; (3) previous gastrointestinal surgery; and (4) unwillingness to undergo LSG. The patients underwent follow-up at 3, 6, and 12 months after LSG (hereafter referred to as 3, 6, and 12 M post-LSG, respectively). This study was conducted in accordance with the Declaration of Helsinki and French Legislation, and all the patients provided informed consent. The study protocol was approved by the Ethics Committee of our hospital (clinical registration number ChiCTR-OCS-12002381).

### Anthropometric and biochemical measurements

Demographic and clinical data, including medical history and date of birth, were analyzed. Anthropometric measurements were performed for all patients. Height and weight measurements were performed by the medical staff, with the patients clad in light clothes, using an Omron HBF-358 body fat analyzer (Q40102010L01322F, Japan). The BMI was calculated as weight in kilograms divided by height in meters squared (kg/m^2^). Morning venous blood was obtained from all patients after a 12h overnight fast and centrifuged thereafter with the supernatant being used in the laboratory tests. Serum FABP1 levels were measured using an enzyme-linked immunosorbent assay (ELISA; Abcam (Cambridge), ab218261) kit. As per the manufacturer’s protocol, the normal reference level of FABP1 was 6.36–19.0 ng/mL. UA, fasting blood glucose (FBG), fasting insulin (FINS), fasting C-peptide (FCP), alanine aminotransferase (ALT), aspartate aminotransferase (AST), and γ-transaminase (γ-GT) levels were assessed using an automated biochemical analyzer.

### Statistical analysis

Statistical analysis was performed using SPSS 20.0 (SPSS Inc., Chicago, IL, USA) and GraphPad Prism 8 (GraphPad Software, San Diego, USA) in Windows 10. Data are presented as means ± standard deviations (SDs) for continuous variables, and count data are expressed as the number of columns (n). Comparisons between continuous or categorical variables in two groups were tested using the Student’s t test or variance analysis, whereas paired-sample t tests were used to compare the data before and after surgery. To explore the correlation between serum FABP1 levels and UA levels or other biochemical criteria, Spearman’s correlation analysis was performed. To further study the relationship between serum FABP1 levels and UA levels, we performed a multiple linear regression. We then performed a binary logistic regression for Models 1, 2, and 3 to identify potential factors related to the risk of hyperuricemia in patients with obesity. Statistical significance was set at *P* < 0.05.

## Results

### Elevated FABP1 levels in the HUA group with obesity

We divided the patients with obesity (male, n = 47 and female, n = 58) into the NUA (n = 44) and HUA (n = 61) groups. There was no statistical difference in the sex distribution between the two groups (*P* = 0.604). The FABP1 levels were found to be significantly higher in the HUA group than in the NUA group at baseline (34.76 ± 22.69 ng/mL vs. 25.21 ± 21.68 ng/mL, *P* = 0.024). Furthermore, the FINS, FCP, ALT, AST and γ-GT levels were also significantly lower in the NUA group than in the HUA group (25.61 ± 20.99 mU/L vs. 37.50 ± 21.61 mU/L, *P* = 0.015; 3.89 ± 1.68 ng/ml vs. 4.97 ± 1.65 ng/ml, *P* = 0.004; 45.69 ± 44.99 U/L vs. 67.67 ± 62.02 U/L, *P* = 0.036; 28.67 ± 23.28 U/L vs. 44.00 ± 36.47 U/L, *P* = 0.013; 44.26 ± 32.47 U/L vs. 62.75 ± 48.00 U/L, *P* = 0.034). However, BMI and FBG levels were not statistically different between the two groups (37.58 ± 6.46 kg/m^2^ vs. 38.98 ± 6.23 kg/m^2^, *P*=0.679; 6.85 ± 3.09 mmol/L vs. 5.80 ± 2.08 mmol/L, *P*=0.054) (**Table 1**)

**Table 1:** Baseline characteristics of the study.

Parameters	Obese population			*P value*
	Total (n = 105)	NUA (n = 44)	HUA (n = 61)	
FABP1 (ng/mL)	30.76 ± 21.53	25.21 ± 21.68	34.76 ± 22.69	0.024*
UA(μmol/L)	420.79 ± 97.65	339.44 ± 49.95	479.47 ± 79.91	0.000***
Age (years)	30.79 ± 10.92	32.47 ± 11.98	29.57 ± 10.01	0.181
Sex(male/female)	47/58	21/23	26/35	0.604
BMI (kg/m^2^)	38.39 ± 6.33	37.58 ± 6.46	38.98 ± 6.23	0.679
FBG (mmol/L)	6.24 ± 2.59	6.85 ± 3.09	5.80 ± 2.08	0.054
FINS(mU/L)	33.02 ± 22.03	25.61 ± 20.99	37.50 ± 21.61	0.015*
FCP(ng/ml)	4.55 ± 1.74	3.89 ± 1.68	4.97 ± 1.65	0.004**
ALT (U/L)	58.46 ± 55.71	45.69 ± 44.99	67.67 ± 62.02	0.036*
AST (U/L)	37.90 ± 32.63	28.67 ± 23.28	44.00 ± 36.47	0.013*
γ−GT (U/L)	54.82 ± 42.85	44.26 ± 32.47	62.75 ± 48.00	0.034*

Data are presented as means ± SDs.

FABP1, liver fatty acid-binding protein; UA, uric acid; BMI, body mass index; FBG, fasting blood glucose; FINS, fasting insulin; FCP, Fasting C-peptide; ALT, alanine aminotransferase; AST, aspartate aminotransferase; γ-GT, γ-transaminase; *P < 0.05, **P < 0.01, ***P < 0.001.

### Correlation between serum FABP1 levels and UA levels at baseline

**Figure 1:**
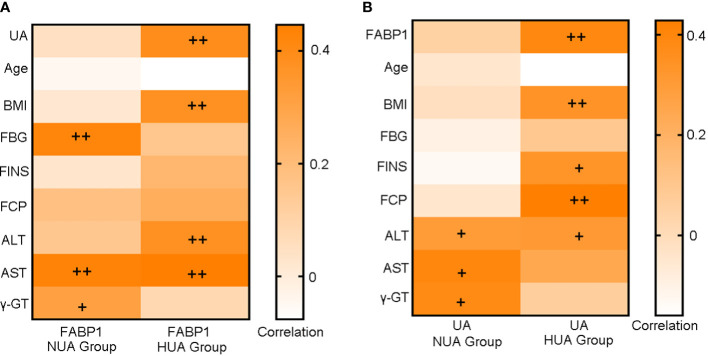
Heat map of the correlation analysis of serum FABP1 levels **(A)** or UA levels **(B)** with glucose metabolism and liver function-related indicators at baseline. +*P*<0.05; ++*P*<0.01.

Since hyperuricemia and FABP1 are closely related to diabetes and liver-related metabolic disorders, we first assessed the correlation between serum FABP1 or UA levels with glucose metabolism and liver enzyme indices in the NUA and HUA groups (**Figure 1**). We found the serum FABP1 levels were related to the FBG, AST and γ -GT levels in the NUA group (r = 0.422, *P* = 0.004; r=0.430, *P* = 0.006; r = 0.311, *P* = 045, respectively). Moreover, the UA levels correlated with the ALT, AST, and γ-GT levels (r = 0.297, *P* = 0.049; r = 0.394, *P* = 0.013; r = 0.381, *P* = 0.013, respectively). Furthermore, in the HUA group, the serum FABP1 level was positively correlated with the UA level (r = 0.390, *P* = 0.002), and with the BMI, ALT and AST levels (r = 0.375, *P* = 0.002; r = 0.376, *P* = 0.003; r = 0.446, *P* < 0.001, respectively). In addition, the UA level were related to the BMI, FINS, FCP and ALT levels (r = 0.338, *P* = 0.008; r = 0.334, *P* = 0.014; r = 0.428, *P* = 0.01; r = 0.308, *P* = 0.016, respectively).

To evaluate the correlation between serum UA levels and FABP1 levels, we also performed a multiple linear regression analysis on all individuals. First, Model 1 revealed a significantly positive correlation between serum UA levels and FABP1 levels (β = 0.344, *P <*0.001). Second, in Model 2, a correlation was also evident after adjusting for BMI, age, and sex (β = 0.257, *P* = 0.005). Third, in Model 3 (β = 0.280, *P* = 0.005), serum FABP1 levels were another determinant of the UA levels after adjusting for BMI, age, and the related indicators of glucose metabolism (FBG, FINS, and FCP). The same findings were observed in Model 4 (β = 0.283, *P* = 0.009) after adjusting for BMI, age, sex, and ALT, AST, and γ-GT levels (**Table 2**).

**Table 2:** Multiple Regression Modeling of the serum UA levels associated with the FABP1 levels.

Model	UA			
	R^2^	β	*P* value	95% CI of β
1	0.118	0.344	0.000***	0.726-2.391
2	0.263	0.257	0.005**	0.361-1.968
3	0.423	0.280	0.005**	0.407-2.224
4	0.311	0.283	0.009**	0.373-2.508

Model 1: FABP1; Model 2: FABP1, adjusting for BMI, age, and sex; Model 3: FABP1, adjusting for BMI, age, sex, and FBG,FINS, and FCP levels; Model 4: FABP1, adjusting BMI, age, sex, and ALT,AST and γ-GT levels; FABP1, liver fatty acid-binding protein; UA, uric acid; BMI, body mass index; FBG, fasting blood glucose; FINS, fasting insulin; FCP, Fasting C-peptide; ALT, alanine aminotransferase; AST, aspartate aminotransferase; γ-GT, γ-transaminase; **P < 0.01, ***P < 0.001.

### Preoperative FABP1 may be a risk factor for hyperuricemia

We also constructed a multivariable logistic model for the occurrence of hyperuricemia in the preoperative obese cohort (**Table 3**). In Model 1, with BMI and age as additional covariables, we observed a statistically significant association between serum FABP1 levels and the risk of hyperuricemia in patients with obesity (Odds ratio [OR], 95% confidence interval [CI] = 1.023, 1.001–1.046, *P* = 0.048). This association was also significant in Model 2 (OR, 95% CI = 1.045, 1.009–1.083, *P* = 0.016) with the same variables as in Model 1 and with FBG, FINS, and FCP levels as additional covariables. After further adjustment for age, BMI, sex, ALT, AST, and γ-GT levels in Model 3, there was no statistical difference in the association between FABP1 and the risk of hyperuricemia (OR, 95% CI = 1.022, 0.994–1.052, *P* = 0.125).

**Table 3:** Multivariable-adjusted association of serum FABP1 levels and hyperuricemia in patients with obesity.

Model	OR (95% CI)	*P* value
1	1.023 (1.001-1.046)	0.048*
2	1.045 (1.009-1.083)	0.016*
3	1.022 (0.994-1.052)	0.125

Model 1:included the serum FABP1 level, age, BMI, and sex; Model 2:included serum FABP1 level, age, BMI, sex, and FBG, FINS, and FCP levels; Model 3: included serum FABP1 level, age, BMI, sex, and ALT,AST and γ -GT levels. FABP1, liver fatty acid-binding protein; UA, uric acid; BMI, body mass index; FBG, fasting blood glucose; FINS, fasting insulin; FCP, Fasting C-peptide; ALT, alanine aminotransferase; AST, aspartate aminotransferase; γ-GT, γ-transaminase; *P < 0.05.

### Correlation between the change in serum UA levels and FABP1 levels after LSG

**Figure 2:**
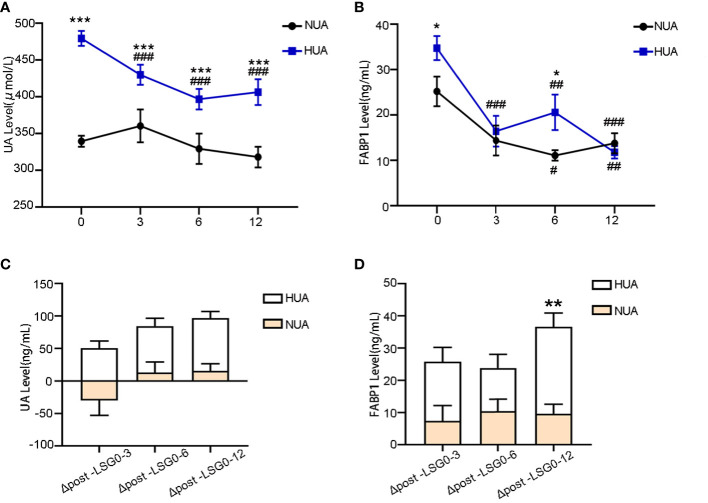
Changes in the serum FABP1 and UA levels between the NUA and HUA groups after LSG. **(A, B)** Decrease in the UA and FABP1 levels after LSG. **(C, D)** Comparison of the magnitude of the change in the UA and FABP1 levels at 3, 6, and 12 M post-LSG and baseline; Comparison of the variables between 3, 6, and 12 M post-LSG and baseline, ^##^ *P* < 0.01, ^###^ *P* < 0.001 Comparison of the variables between the NUA and HUA groups, *P < 0.05, **P < 0.01, ***P < 0.001.

The UA levels decreased significantly in the HUA group after LSG (from 479.47 ± 79.91 µmol/L to 429.98 ± 90.52 µmol/L at 3 M, *P* < 0.001; 396.78 ± 80.87 µmol/L at 6 M, *P* < 0.001; and 406.34 ± 93.90 µmol/L at 12 M, *P* < 0.001) (**Figure 2A**). Moreover, the serum FABP1 levels decreased progressively at 3, 6, and 12 months after surgery (for the NUA group: from 25.21 ± 21.68 ng/mL to 14.38 ± 11.88 ng/mL at 3M, *P* = 0.142 ; 11.09 ± 4.25 ng/mL at 6 M, *P* = 0.015; and 13.77 ± 9.94 ng/mL at 12 M, *P*=0.005; and for the HUA group: from 34.76 ± 22.69 ng/m to 16.43 ± 8.59 ng/mL at 3 M, *P* < 0.001; 20.60 ± 16.62 ng/mL at 6 M, *P* = 0.012; 11.77 ± 6.9 ng/mL at 12 M, *P* < 0.001)(**Figure 2B**). Furthermore, the UA levels in the HUA group showed a greater decrease than that in the NUA group (**Figure 2C**). Meanwhile, the serum FABP1 levels showed a more significant decrease in the HUA group than in the NUA group at 12 months (27.06 ± 10.98 ng/mL vs. 9.54 ± 6.52 ng/mL, *P* = 0.003) (**Figure 2D**). **Table 4** shows the correlations of the changes (△) in the serum FABP1 levels and the metabolic factors in the NUA and HUA groups. The changes in the serum FABP1 levels at 12 months after surgery were positively correlated with the changes in the UA levels (r = 0.512, *P* = 0.011). Moreover, the changes in BMI (r = 0.399, *P* = 0.048) and ALT levels (r = 0.390, *P* = 0.031) were associated with the change in serum FABP1 levels at 12M post-LSG in the HUA group.

**Table 4:** Correlation analysis between the improvement in the serum FABP1 levels and the clinical indices between the different UA groups after LSG.

Parameters	ΔFABP1-3		ΔFABP1-6		ΔFABP1-12	
	r	*P* value	r	*P* value	r	*P* value
**NUA group**						
ΔUA	-0.401	0.174	0.214	0.068	0.211	0.386
ΔBMI	0.231	0.412	-0.325	0.257	0.166	0.448
ΔFBG	0.476	0.100	0.345	0.010**	-0.094	0.670
ΔFINS	-0.248	0.414	0.398	0.159	-0.191	0.383
ΔFCP	-0.272	0.368	0.312	0.541	-0.245	0.260
ΔALT	0.021	0.946	0.421	0.134	0.180	0.460
ΔAST	0.029	0.926	0.341	0.049*	0.155	0.537
Δγ−GT	-0.148	0.683	0.378	0.135	0.123	0.639
**HUA group**						
ΔUA	0.198	0.304	0.318	0.214	0.512	0.011*
ΔBMI	-0.141	0.465	0.557	0.020*	0.399	0.048*
ΔFBG	0.260	0.173	0.139	0.595	0.175	0.364
ΔFINS	0.104	0.591	0.131	0.617	0.241	0.225
ΔFCP	0.171	0.376	0.235	0.364	0.223	0.254
ΔALT	0.346	0.071	0.378	0.135	0.390	0.031*
ΔAST	0.436	0.020	0.347	0.173	0.266	0.220
Δγ−GT	0.123	0.575	0.140	0.632	0.133	0.566

FABP1, liver fatty acid-binding protein; UA, uric acid; BMI, body mass index; FBG, fasting blood glucose; FINS, fasting insulin; FCP, Fasting C-peptide; ALT, alanine aminotransferase; AST, aspartate aminotransferase; γ-GT, γ-transaminase; *P < 0.05, **P < 0.01.

To evaluate the contribution of FABP1 and other metabolic factors toward UA, a multiple linear regression analysis was also performed in **Table 5**. After adjusting the △BMI, age, and sex, △FBG, △FINS, △FCP, △ALT, △AST and △γ-GT levels, ΔUA (at 12M post-LSG) was also significantly correlated with ΔFABP1.

**Table 5:** Multiple linear analysis of the correlation between changes in UA and FABP1 levels at 12M post-LSG.

Model	△UA			
	R^2^	β	*P* value	95% CI of β
1	0.287	0.536	0.000***	0.352-2.379
2	0.423	0.458	0.002**	0.573-2.237
3	0.436	0.470	0.002**	0.547-2.343
4	0.471	0.550	0.001**	0813-3.025

Model 1: △FABP1;

Model 2: △FABP1, adjusting for △BMI, age, and sex;

Model 3: △FABP1, adjusting for △BMI, age, sex, and △FBG, △FINS, and △FCP levels;

Model 4: △FABP1, adjusting △BMI, age, sex, and △ALT, △AST and △γ-GT levels;

FABP1, liver fatty acid-binding protein; UA, uric acid; BMI, body mass index; FBG, fasting blood glucose; FINS, fasting insulin; FCP, Fasting C-peptide; ALT, alanine aminotransferase; AST, aspartate aminotransferase; γ-GT, γ-transaminase; **P < 0.01,***P < 0.001.

## Discussion

Currently, FABP1 is commonly used as a specific biomarker for liver disease and type 1 diabetes mellitus in clinical practice (13–15). However, hyperuricemia as a common metabolic disease is closely related to liver and pancreas islet function, and its relationship with FABP1 has not been reported. Our study demonstrated that serum FABP1 levels were significantly higher in the HUA group than in the NUA group and the positive correlativity between the change in FABP1 and UA levels at 3, 6, and 12 months after LSG. To the best of our knowledge, this is the first study to provide clinical evidence confirming the relationship between serum FABP1 levels and hyperuricemia in patients with obesity before and after LSG.

Hyperuricemia refers to a disorder of purine metabolism or decreased UA excretion, leading to increased SUA levels. Studies (16, 17) have shown that this disorder forms not only the biochemical basis for gout but can also induce major diseases, such as myocardial infarction, diabetes, coronary heart disease, metabolic syndrome, and other diseases, which ultimately deprive patients’ lives. The meta-analysis (18) suggested that the OR value of hypertension risk in HUA patients was 1.48, especially in younger patients with early-onset hypertension. Besides, clinical studies (19, 20) have found that elevated SUA at baseline can predict the incidence of diabetes and insulin resistance (IR) status, suggesting that elevated SUA is an independent risk factor for IR and diabetes. Our study also found that compared with the NUA group, patients in the HUA group had relatively higher BMI and poorer pancreatic islet and liver function. These results suggest the danger of hyperuricemia and the urgency of treatment. Thus, there was an urgent need for further studies to identify the pathogenesis of hyperuricemia.

FABPs (21–23) belong to the lipid-binding protein superfamily. To date, nine types of FABPs have been identified based on tissue-specific distributions: L (liver), I (intestinal), H (muscle and heart), A (adipocyte), E (epidermal), Il (ileal), B (brain), M (myelin), and T (testis) (24). The first described FABP, liver-FABP (L-FABP or FABP1), is expressed highly in the liver, as well as in the intestines and kidneys. FABP1 comprises 127 amino acids with a molecular weight of approximately 14–15 kDa, can regulate the expression of some essential genes involved in lipid metabolism, and is related closely to a variety of metabolic syndromes, such as obesity, NAFLD, and IR (25, 26). In our previous study, we also found that serum FABP1 levels positively correlated with BMI, and after performing a multiple linear regression adjusted for sex, age, ALT, and other factors, the serum FABP1 levels remained strongly correlated with BMI (10). Newberry (27) found that, compared with wild-type mice, the *FABP1*-gene knockout mice could significantly inhibit diet-induced obesity and reduce the development of fatty liver when fed a diet rich in fatty acids. This was related to the reduced absorption of fatty acids and their esterification in the intestines of mice with this gene deletion, which affects the synthesis rate of chylomicrons. Therefore, serum FABP1 levels can change a variety of metabolic factors and regulate the pathogenesis of obesity.

Furthermore, FABP1 is closely related to the occurrence and development of other metabolic syndromes. Studies have found that FABP1 plays an important role in lipid transport and cholesterol metabolism in the liver. *FABP1* expression increased significantly with the formation of fatty liver and was correlated with the degree of hepatic steatosis, proving that serum FABP1 levels play an important role in NAFLD using a mouse model of NAFLD (28). Moreover, the serum FABP1 levels are related to glucose metabolism. Shi Juan et al. (29) found that serum FABP1 levels increased in adolescents with abnormal glucose metabolism and were related to glucose and lipid metabolism but not to the function of pancreatic islet β cells. Furthermore, a previous study suggested that the serum FABP1 level in the renal tubules is associated with glomerular disease and FABP1 plays a protective role in renal tubulointerstitial injury and glomerular injury of diabetic nephropathy (30). However, the relationship between FABP1 and hyperuricemia is unknown. In this study, we also found that serum FABP1 levels were closely related to UA, liver enzyme and glucose metabolism parameters; moreover, it is worth highlighting that after adjusting for confounding factors, preoperative FABP1 level was a risk factor for hyperuricemia at baseline.

Bariatric surgery has been found to regulate BMI safely and effectively and improve metabolic markers such as blood glucose and liver function levels. In recent years, multiple pieces of evidence (31–33) have shown that bariatric surgery is effective in reducing the incidence of gout attacks and serum urate concentration in patients with hyperuricemia or gout up to 12 months of follow-up. Our results also indicated UA and FABP1 levels decreased at 3, 6, and 12 months postoperatively, and the decrease was greater in the HUA group than in the NUA group at 3, 6, and 12 months after LSG; Moreover, the changes in the serum FABP1 levels at 12 months after LSG showed a positive correlation with changes of SUA levels in the HUA group.

The current study applied a variety of statistical methods to elucidate the relationship between serum FABP1 levels and hyperuricemia before and after LSG for the first time. However, our study had certain limitations. First, owing to the observational nature of this study, we could not determine any causal relationship between the UA levels and elevated serum FABP1 levels. Moreover, this study did not investigate the mechanism in depth. In binomial logistic regression, ​we found that the association between serum FABP1 levels and the risk of hyperuricemia had no statistical effect after adjusting for liver enzymes. Now, a large number of studies have shown that UA levels are closely related to liver diseases, such as NAFLD (34–36), and are also positively correlated with liver enzyme indicators (37). And there is no doubt that IR must play a crucial role in this connection. FABP1 is also mainly expressed in the liver. Many studies have also proved the association between FABP1 and liver diseases (15, 38). Silencing of FABP1 can ameliorate hepatic steatosis, inflammation, and oxidative stress (38). And FABP1 can be used as a marker of liver damage after medication (15). Meanwhile, FABP1 is also positively correlated with liver enzymes levels. So we speculated that FABP1 and hyperuricemia may interact through liver function-related indicators. We will conduct further research using animal and cell experiments in the future. Second, the study had a small sample size and short follow-up durations. There was a sampling error in this study. Future studies with larger numbers of patients and longer follow-up periods are warranted to validate our findings.

## Conclusions

Serum FABP1 levels were significantly higher in the HUA group than in the NUA group. In addition, serum FABP1 levels positively correlated with UA levels, and the preoperative serum FABP1 levels may be a risk factor for hyperuricemia. Moreover, the serum FABP1 levels decreased postoperatively and with a greater reduction in the HUA group than in the NUA group at 3, 6, and 12 M after LSG. Furthermore, changes in the serum FABP1 levels at 12 M after LSG showed a positive correlation with changes in the UA levels in the HUA group. Our findings may provide a basis for encouraging researchers and clinicians working on metabolic diseases to adopt a new perspective on the role of FABP1, and endorse FABP1 as an important indicator of hyperuricemia.

## Data availability statement

The original contributions presented in the study are included in the article/supplementary material. Further inquiries can be directed to the corresponding authors.

## Ethics statement

The studies involving human participants were reviewed and approved by ClinicalTrial.gov ID: ChiCTR-OCS-12002381. The patients/participants provided their written informed consent to participate in this study.

## Author contributions

JZ and SQ conceived and supervised the overall study. XW, CZ, WM, LB, MZ, JY, and LD performed the literature review and collected the epidemiological and clinical data. XC and HC contributed to the statistical analysis. HY, HM, and XCW drafted the manuscript. All authors contributed to the article and approved the submitted version.

## Funding

This work was supported by the National Natural Science Foundation of China (grant numbers 81700752, 81970677, 82170861, 82170904), Traditional Chinese Medicine Scientific Research Project of Shanghai Municipal Health Commission (grant number 2020_JP013), and Climbing Talent Program of the 10th People's Hospital affiliated to Tongji University (2021SYPDRC059,2021SYPDRC050).

## Conflict of interest

The authors declare that the research was conducted in the absence of any commercial or financial relationships that could be construed as a potential conflict of interest.

## Publisher’s note

All claims expressed in this article are solely those of the authors and do not necessarily represent those of their affiliated organizations, or those of the publisher, the editors and the reviewers. Any product that may be evaluated in this article, or claim that may be made by its manufacturer, is not guaranteed or endorsed by the publisher.
